# Effectiveness of trigger point dry needling for plantar heel pain: study protocol for a randomised controlled trial

**DOI:** 10.1186/1757-1146-4-5

**Published:** 2011-01-23

**Authors:** Matthew P Cotchett, Karl B Landorf, Shannon E Munteanu, Anita Raspovic

**Affiliations:** 1 Department of Podiatry, Faculty of Health Sciences, La Trobe University, Bundoora, 3086, Australia; 2 Musculoskeletal Research Centre, Faculty of Health Sciences, La Trobe University, Bundoora, 3086, Australia

## Abstract

**Background:**

Plantar heel pain (plantar fasciitis) is a common and disabling condition, which has a detrimental impact on health-related quality of life. Despite the high prevalence of plantar heel pain, the optimal treatment for this disorder remains unclear. Consequently, an alternative therapy such as dry needling is increasingly being used as an adjunctive treatment by health practitioners. Only two trials have investigated the effectiveness of dry needling for plantar heel pain, however both trials were of a low methodological quality. This manuscript describes the design of a randomised controlled trial to evaluate the effectiveness of dry needling for plantar heel pain.

**Methods:**

Eighty community-dwelling men and woman aged over 18 years with plantar heel pain (who satisfy the inclusion and exclusion criteria) will be recruited. Eligible participants with plantar heel pain will be randomised to receive either one of two interventions, (i) real dry needling or (ii) sham dry needling. The protocol (including needling details and treatment regimen) was formulated by general consensus (using the Delphi research method) using 30 experts worldwide that commonly use dry needling for plantar heel pain. Primary outcome measures will be the pain subscale of the Foot Health Status Questionnaire and "first step" pain as measured on a visual analogue scale. The secondary outcome measures will be health related quality of life (assessed using the Short Form-36 questionnaire - Version Two) and depression, anxiety and stress (assessed using the Depression, Anxiety and Stress Scale - short version). Primary outcome measures will be performed at baseline, 2, 4, 6 and 12 weeks and secondary outcome measures will be performed at baseline, 6 and 12 weeks. Data will be analysed using the intention to treat principle.

**Conclusion:**

This study is the first randomised controlled trial to evaluate the effectiveness of dry needling for plantar heel pain. The trial will be reported in accordance with the Consolidated Standards of Reporting Trials and the Standards for Reporting Interventions in Clinical Trials of Acupuncture guidelines. The findings from this trial will provide evidence for the effectiveness of trigger point dry needling for plantar heel pain.

**Trial registration:**

Australian New Zealand 'Clinical Trials Registry'. ACTRN12610000611022.

## Background

Plantar heel pain (plantar fasciitis) is one of the most common musculoskeletal pathologies of the foot. It is estimated to effect 10% of the population at some time in their life [1], although there are few high quality epidemiological studies available. One national study of medical doctors in the United States during the years 1995 to 2000 found that approximately one million patient visits to physicians or hospital outpatient departments per year were for plantar heel pain [2] at a projected cost of between $US192 to $US376 million dollars to third - party payers [3]. In addition, a recent Australian study of 3206 adults found that approximately 20.9% indicated that they had heel pain, although this study did not differentiate between plantar heel pain and pain in other parts of the heel [4].

It is generally accepted that plantar heel pain predominantly affects middle aged as well as older adults. In a study of 784 North American community dwelling residents aged 65 years or greater, 7% reported pain and tenderness beneath the heel [5]. Although plantar heel pain affects older adults it is also common in the athletic population, being estimated to contribute to 25% of all foot injuries related to running [6]. Plantar heel pain has been shown to have an impact on health-related quality of life. A recent case control study found that individuals with chronic plantar heel pain are severely limited in their ability to undertake physical activities and lack the energy to undertake daily tasks, have a poor perception of their health status and experience social isolation [7].

A dearth of facts and an abundance of opinions surround the optimal treatment of plantar heel pain. Despite its prevalence [2,4], financial burden [3] and detrimental impact on health-related quality of life [7], evidence-based clinical practice guidelines for plantar heel pain [8] do not recommend one treatment over another. In addition, two systematic reviews [1,9] have found few interventions that are supported by good quality evidence.

An alternative treatment for plantar heel pain is trigger point dry needling, which involves stimulation of myofascial trigger points (MTrPs) using a fine filament needle. Dry needling is increasingly used by physical therapists [10] for the treatment of neck pain [11], shoulder pain [12], knee pain [13], posterior thigh pain [14] and low back pain [15-17].

Although MTrP dry needling is becoming increasingly used for the treatment of plantar heel pain, only two studies have been published that have investigated the effectiveness of this intervention for this disorder [18,19]. Tillu and Gupta [18] found a significant improvement in plantar heel pain, as measured on a visual analogue scale (67.9% improvement, p = 0.047), with a four-week (one treatment per week) period of acupuncture followed by two weeks of dry needling of the calf and heel regions. Perez-Milan and Foster [19] also demonstrated a significant reduction in pain (46% improvement, p < 0.001) with a six-week (one treatment per week) program of acupuncture and dry needling of the heel and arch. However, the quality of these trials as measured by the Quality Index [20] was poor and therefore the positive effects of the MTrP treatment are likely to have been overestimated [21]. For example, both trials did not have a control comparison and there was no evidence of blinding of the outcome assessors. Also of importance was the absence of information detailing the criteria used to diagnose a MTrP and the specific location of MTrPs that were dry needled.

In light of limitations of previous studies [18,19] mentioned above, the aim of this project is to investigate whether deep trigger point dry needling is more effective than sham (non-insertive simulated) dry needling for plantar heel pain. The proposed project will utilise rigorous randomised controlled methodology. The study protocol for the proposed randomised controlled trial presented in this article is consistent with the recommendations of BioMed Central [22].

## Methods

### Design

This study is a parallel-group participant and assessor blinded, randomised controlled trial. The trial has been registered on the Australian New Zealand 'Clinical Trials Registry' (ACTRN12610000611022) - a requirement by the International Committee of Medical Journal Editors. The trial will be reported in line with the Consolidated Standards of Reporting Trials (CONSORT) [23] and the Standards for Reporting Interventions in Clinical Trials of Acupuncture (STRICTA) [24] group statements.

**Figure 1:**
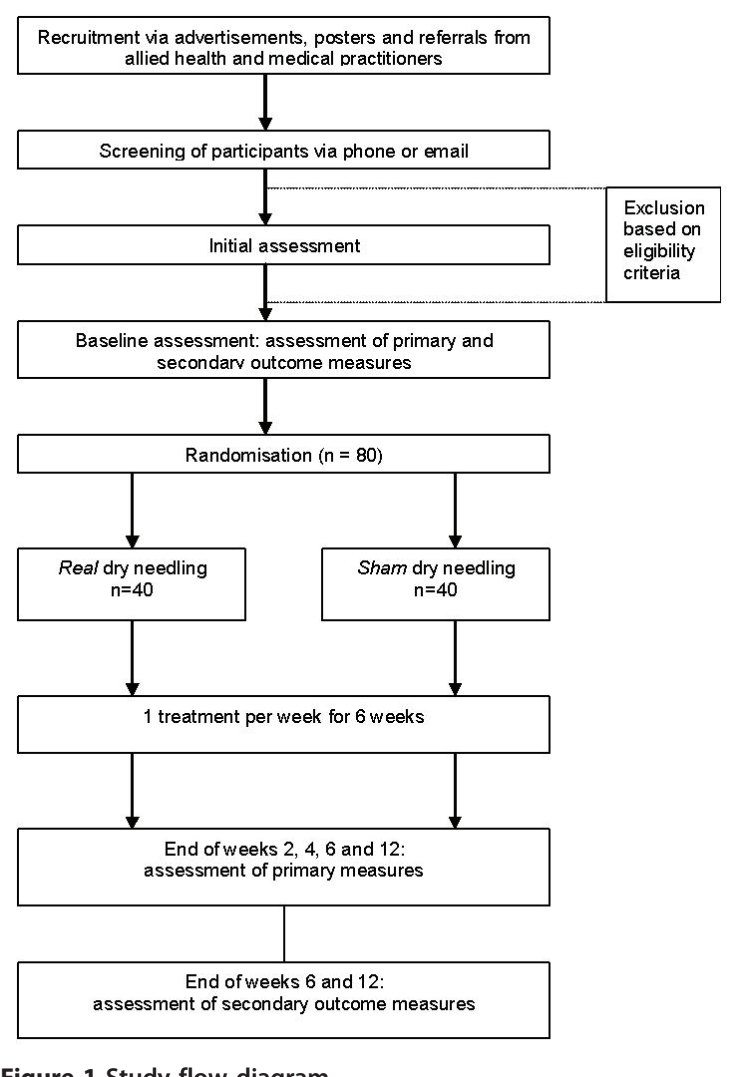
**Study flow diagram**.

Participants will be randomised to receive either *real *dry needling or a *sham *dry needling intervention. Allocation to either the real or sham groups will be achieved using a computer generated random number sequence The allocation sequence will be generated and held by an external person (an administrative officer in the Department of Podiatry, La Trobe University) not directly involved in the trial. Importantly, this person will not be present at recruitment, will have no participant contact and will not be involved in collection and processing of data collected during the trial. The allocation sequence will be concealed from the researcher (MC) enrolling and assessing participants as each participant's allocation will be contained in sequentially numbered sealed and stapled opaque envelopes. In addition, a system using carbon paper will be employed so the participants' details (name and date of recruitment) are transferred from the outside of the envelope to the paper inside the envelope containing the allocation prior to opening the seal. This method of allocation concealment has been used previously [25,26] and has been recommended by the CONSORT group http://www.consort-statement.org/consort-statement/3-12---methods/item9_randomisation-allocation-concealment-mechanism/. Figure 1 shows a flow diagram of the progress through the different phases of this trial. Ethics approval has been obtained from La Trobe University's Faculty Human Ethics Committee (No.10-015).

### Participants

Participants with plantar heel pain that provide informed consent will be recruited from the local community via:

i) Advertisements in local and greater Melbourne newspapers;

ii) Mail-out advertisements to local medical and allied health practitioners;

iii) Advertisements on relevant internet sites;

iv) Posters displayed in local community centres, sporting clubs, retirement villages, Melbourne universities;

v) Advertisements on Melbourne radio.

People interested in the study will be instructed to contact the Chief Investigator (Mr Matthew Cotchett) via phone or email and will be screened for eligibility. Respondents that are deemed suitable for the study will be invited to attend an initial assessment at the La Trobe University Health Sciences Clinic. To be included in the study, participants must meet the following inclusion criteria:

i) Age greater than 18 years;

ii) Clinical diagnosis of plantar heel pain in accordance with the Clinical Guidelines linked to the International Classification of Function, Disability, and Health from the Orthopaedic Section of the American Physical Therapy Association [8]. The criteria will include:

• Pain in the plantar medial heel region;

• Plantar heel pain that is aggravated by weightbearing activities and worse in the morning and/or upon weightbearing after periods of rest;

• Pain on palpation of the medial calcaneal tubercle.

iii) History of plantar heel pain for greater than one month;

iv) First step pain during the previous week rated at least 20 mm on a 100 mm visual analogue scale;

v) Participants must be willing to attend the La Trobe University Health Sciences Clinic for an initial assessment and then be randomly assigned to receive either the *real *or *sham *intervention. In addition, participants must be willing to receive one treatment per week for a total of six weeks;

vi) A willingness to not receive or implement any form of physical therapy (e.g. foot orthoses, night splints, foot taping, massage therapy and/or footwear modifications) for the duration of the trial;

vii) Be willing to discontinue taking all pain relieving medications (analgesics and non-steroidal anti-inflammatory medications (NSAIDS), except paracetamol up to 4 g/day, taken by mouth or applied topically:

• For at least 14 days prior to the baseline assessment;

• During the study period (6 weeks after the final treatment).

Participants who do take paracetamol need to discontinue its use at least 24 hours prior to the baseline assessment and follow up assessments at 2, 4, 6 and 12 weeks;

viii) An ability to speak read and write English;

ix) An ability to walk 50 metres without the aid of support.

Exclusion criteria for participants will be:

i) Participant refusal to be needled;

ii) The presence of coagulopathy or the use of anti-coagulants (except for acetylsalicylic acid at dosages up to 325 mg/day);

iii) Woman who are pregnant;

iv) Dermatological disease within the dry needling areas;

v) A history of dry needling or acupuncture treatment for any condition;

vi) Treatment for plantar heel pain in the previous 4 weeks;

vii) An inability to understand instructions or complete a questionnaire;

viii) Presence of peripheral arterial vascular disease defined as [27]:

• Failure to palpate at least one pedal pulse and an ankle/brachial index <0.9;

• History of intermittent claudication;

• History of chronic limb ischaemia including rest pain and or lower limb and foot ulceration;

• History of chronic lower limb and foot oedema;

• History of vascular surgery of the lower limb or foot.

ix) History of plantar heel pain secondary to connective tissue disease;

x) The presence of a chronic medical condition that might preclude participation in the study such as: malignancy, systemic inflammatory disorders (e.g., rheumatoid arthritis, psoriatic arthritis, ankylosing spondylitis, septic arthritis), neurological abnormalities, sciatica, and/or chronic pain;

xi) A history of surgery to the plantar fascia;

xii) A history of injection therapy in the heel in the previous three months;

xiii) A known hypersensitivity to metals;

### Interventions

The protocol, including needling details and treatment regimen, was formulated by general consensus (using the Delphi research method) using 30 experts worldwide that commonly use dry needling for plantar heel pain (*unpublished data: Cotchett MP, Landorf KB, Munteanu SE, Raspovic AM: Consensus for dry needling for plantar heel pain (plantar fasciitis): a modified Delphi study. Manuscript submitted for publication*). Participants will be treated by a registered podiatrist (MC) who has 12 years of clinical practice experience and 4 years dry needling experience including 84 hours of dry needling training and 32 hours in dry needling instruction. Table 1 provides a detailed outline of the treatment protocol.

**Table 1:** Dry needling protocol for plantar heel pain, developed by consenus

**Setting**	Treatment will be conducted in the La Trobe University Health Sciences Clinic, Bundoora, Melbourne, Australia.	
**Consultation**	Treatment will be conducted within a 30-minute timeframe. The participant will be lying down.	
**Rationale**	Myofascial trigger point model.	
**Dry needling details**	1.	*Brand of acupuncture needle*: Seirin™ J-Type or Hwa-To™ Ultraclean.
	2.	*Muscles to be dry needled*. Muscles to be assessed first will include those harbouring myofascial trigger points that might be responsible for the participant's pain including the Sol, QP, FDB and Abd H muscles. Synergists and antagonists of these muscles will also be assessed for MTrPs. These muscles will include the Gastroc, FDL, FHL, PL, PB, TA, EHL, EDL, Add H, Abd Dig Min, Lb and Int. In addition a search will be undertaken for MTrPs in muscles which might be influencing the participant's loading of the aforementioned muscles. These muscles will include the Pf, G Max, G Med, G Min, TFL, AL, AM, AB, ST, SM and BF.
	3.	*Needle length and diameter*. Needle length will be determined by the location of the MTrP to be dry needled. Most commonly the needle length will range from 30 to 75 mm. The diameter of the needle will be 0.30 mm but will be varied depending on the participant's tolerance to insertion of the needle. A smaller diameter needle may be used if needle insertion is uncomfortable.
	4.	*Needle insertions per muscle*. The number of needle insertions per muscle will depend on: the number of MTrPs to be dry needled; participant's tolerance to needle insertion; responsiveness of the tissue to dry needling; and level of post needle soreness for a specific muscle. Most commonly the number of needle insertions will range from 1-5.
	5.	*Response elicited*. Dry needling of a MTrP will attempt to elicit an appropriate response such as a: local twitch response (LTR); sensation such as a dull ache, heaviness, distension, pressure or bruising; and/or a reproduction of the participant's symptoms. If an appropriate response is not elicited the needle will be removed and the participant re-examined.
	6.	*Manipulation of the acupuncture needle*. Following insertion, the acupuncture needle will be withdrawn partially and advanced repeatedly to produce an appropriate response. If the participant is sensitive to insertion of the needle the manipulation will be reduced. If this action is insufficient to reduce the painful stimulus, the manipulation will be ceased and the needle left in situ. Alternatively, the needle may be replaced with a needle that has a smaller diameter.
	7.	*Needle retention time*. The needle will remain in the muscle for as long as it takes to produce an appropriate response and is tolerated by the participant. Once this has occurred the needle will be left in situ for 5 minutes. This will allow sufficient time for the stimulus to subside in participants that are sensitive to the treatment.
**Treatment regimen**	The clinical trial will involve 1 treatment per week for 6 weeks. Treatment will be ceased if a participant's symptoms resolve prior to the course of the dry needling treatment. However, if a participant experiences a relapse within the 6 week treatment period they will be offered further weekly treatment (s) until the end of the 6 week course.	

**Key: **ADM (abductor digiti minimi); Abd H (abductor hallucis); Add H (adductor hallucis); QP (quadratus plantae); FDB (flexor digitorum brevis); Lb (lumbricales); Int (interossei); Sol (soleus); Gastroc (gastrocnemius); FHL (flexor hallucis longus); FDL (flexor digitorum longus); PL (peroneus longus); PB (peroneus brevis); TA (tibialis anterior); EHL (extensor hallucis longus); EDL (extensor digitorum longus); G MAX (gluteus maximus); G Med (gluteus medius); G Min (gluteus minimus); Pf (piriformis); TFL (tensor fascia latae); AL (adductor longus); AM (adductor magnus); AB (adductor brevis), ST (semitendinosis); SM (semimembranosis) and BF (biceps femoris).

Eligible participants with plantar heel pain will be randomised to receive either one of two interventions, (i) real dry needling or (ii) sham dry needling. In the context of this study, real dry needling, involves stimulation of MTrPs using an acupuncture needle whereas sham dry needling involves simulated dry needling (non-invasive) that is designed to mimic real dry needling treatment being evaluated. Tough et al. [28] found that a non-penetrating sham acupuncture needle was a credible control for dry needling of MTrPs in participants with whiplash associated pain.

If the participant's symptoms are bilateral both sides of the lower extremity will be treated. At the commencement of the treatment, the participant will be lying down. For both interventions, MTrPs will be identified using a list of essential criteria and a list of observations that help confirm the presence of a MTrP [29]. A flat palpation or pincer technique will be used to palpate a MTrP depending on the muscle being assessed [30].

Once the MTrPs have been identified, dry needling will begin. The participant will remain lying down and positioned supine or prone depending on the muscle to be treated. A curtain will be placed at the level of the thoracic spine so that the participant is blinded to needle preparation, needling technique and needle disposal. Cushions will be placed between the participant's legs to help prevent curious participants touching the opposing limb in an attempt to ascertain their treatment allocation.

#### Real dry needling

A detailed explanation of the real dry needling intervention including treatment rationale, dry needling details and treatment regime is outlined in Table 1. A demonstration of the dry needling technique can be found in Additional file 1.

#### Sham dry needling

Sham dry needles have been prepared using a similar method outlined by Tough et al. [28] by removing the tip of the acupuncture needle with wire cutters. A diamond honing stone was then used to polish the end of the acupuncture needle to create a blunt surface. A new sham needle will be sterilised prior to each treatment.

At the commencement of the treatment, a pre-prepared sham acupuncture needle will be removed from its packaging to simulate removal of a real acupuncture needle. The sham needle will be manipulated using the same technique as for the real intervention group [28] - (Additional file 2).

As the sham acupuncture needle is non-penetrating it cannot be left in situ for five minutes as is the case for the real intervention group. Therefore, following five minutes of treatment of each MTrP the Chief Investigator will mimic removal of the needle by placing a finger on either side of the point treated and will pretend to remove the sham acupuncture needle [11,13]. The sham needle and guide tube will be placed into a petri dish but will not be disposed of as it will be required to treat all MTrPs. Instead, a real acupuncture needle will be disposed in a sharps container simulating the noise and effects associated with sharps disposal. This procedure has been used elsewhere [28].

### Participant activity during the trial

A modified pain-monitoring model [31] will be used to guide the amount of activity (such as running and jumping) undertaken by participants during the course of the trial. Under this approach, participants will be permitted to continue any exercise during the trial, however pain is not to exceed level 5 on a 10-point visual analogue scale (VAS). While pain up to level 5 is acceptable, if 'first step' pain (as measured using a VAS) increases from one week to the next the participant will be advised to lower the level of exercise. The pain monitoring model has been used to guide the rehabilitation of patients with patellofemoral pain syndrome [32] and achilles tendinopathy [31,33].

### Controlling non-specific effects associated with dry needling

To ensure non-specific effects (i.e. those effects that may be observed that are not directly related to the intervention) are controlled, the presentation of the chief investigator; amount of contact time with participants; overall concern and attentiveness directed toward the participants and the manner in which information is presented, will be closely matched. In addition, both groups will be presented with a standardised verbal description of the treatment procedure, which was similarly conducted by Tough et al. [28]. Refer to Additional file 3 for a description of the procedure presented to participants.

### Assessments

#### Initial assessments

During the initial assessment, eligibility of potential recruits will be determined further. At this appointment a range of descriptive characteristics will be also be recorded including: (i) gender, (ii) age, (iii) weight, (iv) height, and (v) hip to waist circumference ratio. Data will also be obtained concerning: (i) duration of symptoms, (ii) side of symptoms, (iii) previous treatment, (iv) type, level and frequency of activity using the 7 - day Physical Activity Recall (PAR) questionnaire [34], (v) foot posture as measured using the Foot Posture Index tool [35], and (vi) the number of MTrPs located within the soleus, abductor hallucis, flexor digitorum and quadratus plantae muscles.

#### Outcome measures

All primary outcome measures will be performed at baseline then 2, 4, 6 and 12 weeks using a blinded assessor (Table 2). Secondary outcome measures will be performed at baseline, 6 and 12 weeks. The primary end-point for predicting the effectiveness of dry needling for plantar heel pain (using the primary outcome measures) will be 6 weeks. All measures will be done prior to any treatment consultation.

**Table 2:** Timeline for primary and secondary outcome measurements

Outcomes						
		**Baseline**	**2 weeks**	**4 weeks**	**6 weeks**	**3 months**

**Primary**						
FHSQ	Pain	✔	✔	✔	✔	✔
VAS	'first-step' pain	✔	✔	✔	✔	✔

**Secondary**						
FHSQ	Function	✔			✔	✔
	General foot	✔			✔	✔
	health					
SF-36		✔			✔	✔
DASS-21		✔			✔	✔

Notes: FHSQ = Foot Health Status Questionnaire; VAS = Visual analogue scale; SF-36 = Short-form-36; CEQ = Credibility/Expectancy Questionnaire; DASS-21 = Depression Anxiety Stress Scale - short version.

#### Primary outcome measures

##### 1. Foot Health Status Questionnaire (FHSQ) - pain

The primary outcome measure will be the pain subscale of the Foot Health Status Questionnaire (FHSQ). The FHSQ has been validated (content, criterion and construct validity) [36]. It has high test-retest reliability (intraclass correlation coefficient ranging from 0.74-0.92) and a high degree of internal consistency (Cronbach's α ranging from 0.85 to 0.88) [36]. It has been used in similar trials that have evaluated the effectiveness of different interventions for plantar heel pain [25,37].

##### 2. Visual analogue scale (VAS) - 'First-step' pain

Severity of pain at the heel when getting out of bed in the morning (also referred to as 'first-step' pain), over the past week, will be assessed using a 100 mm visual analogue scale (VAS). The left side of the scale (0 mm) will be labeled 'no pain' and the right side of the scale (100 mm) will be labelled 'worst pain imaginable'. The VAS is widely used and is valid [38] and reliable [39].

#### Secondary outcome measures

##### 1. Foot Health Status Questionnaire (FHSQ) - foot function and general foot health

Foot function and general foot health will be assessed using the Foot Health Status Questionnaire [36].

##### 2. Short form-36 (SF-36) - health-related quality of life

Health-related quality of life will be assessed using the Short Form-36 version 2 (SF-36). The SF-36 is a 36 item health-related quality of life survey that measures the impact of functional health and well being from the patient's perspective. The SF-36 is widely used and has been extensively validated (concurrent, content, construct, criterion and predictive validity) and has good test-retest reliability [40-42].

##### 3. Depression, Anxiety and Stress Scale short version (DASS-21)

The severity of symptoms of depression, anxiety and stress will be determined using the short version of the Depression, Anxiety and Stress Scale (DASS-21) [43]. The DASS-21 contains 21 items in total that assess the severity of each condition. Participants will be asked to use a 4-point severity/frequency Likert scale to rate the extent to which they have experienced each state over the past week.

Scores for depression, anxiety and stress will be calculated by summing scores for relevant items. High scores on the DASS-21 indicate a high level of distress in the participant. The score for each condition is then evaluated as per the severity-rating index (i.e normal to extremely severe). The DASS-21 has been shown to have high internal consistency and temporal stability [43]. The DASS-21 has been previously validated (content, construct, convergent and discriminative validity) [43,44].

#### Other measures

##### 1. Adverse events

A pre-specified checklist of potential adverse events will be administered so that any adverse event experienced since the previous treatment can be recorded. An open-response type format will also be available for participant responses. Participants will be asked to rate the perceived degree of severity (mild, moderate and severe) for each type of adverse event. In addition, the chief investigator will record adverse events that occur during the treatment.

All adverse events will be classified as non-serious (pain at the site of needle insertion; bleeding; feeling faint; drowsiness; nausea; sweating; infection; needle allergy; exacerbation of symptoms) or serious (any adverse event that leads to serious disability; hospital admission; is life threatening or results in death) as defined by Australia's Therapeutic Goods Administration [45]. A detailed description will be made of any adverse event that results in withdrawal of participants from the trial.

##### 2. Use of rescue medication to relieve plantar heel pain

Participants will be required to complete a medications diary to record the type and amount of rescue medication consumed for their plantar heel pain. Rescue medication is defined as medication (e.g. paracetamol) participants can use during the study, if required. The diary will be returned to the Chief Investigator at 6 and 12 weeks. The number of participants that consume rescue medication and the average amount of medication (mean grams of paracetamol/participant/month) [26,33] will be determined.

##### 3. Use of co-interventions to relieve plantar heel pain

Participants will also be asked to complete a diary to outline other treatments they received during the trial period to help relieve their plantar heel pain. Such treatments include oral non-steroidal anti-inflammatories, topical medicaments (such as rubefacients, or topical non-steroidal anti-inflammatories), foot orthoses, night splints, calf stretching, massage therapy, footwear modifications, foot taping, foot injections [33,46]. Participants will also be questioned to determine if they have changed their footwear during the course of the trial. The diary will be returned to the Chief Investigator at 6 and 12 weeks.

##### 4. Seven day Physical Activity Recall (PAR) - level of physical activity in the previous week

The level of activity in the previous week will be evaluated using the 7-day Physical Activity Recall (PAR) questionnaire [34]. The PAR questionnaire estimates the amount of time the participant spent in physical activity, strength and flexibility activities in the seven days prior to completing the questionnaire. The PAR only records physical activity of moderate or greater intensity. The frequency and duration of each activity undertaken is combined with its metabolic equivalent value to calculate the total kilocalories of energy expenditure per day for each participant. The PAR has been shown to have good reliability and validity [34].

##### 5. Credibility/Expectancy Questionnaire (CEQ)

The Credibility/Expectancy Questionnaire (CEQ) [47] will be administered after the first treatment only, which provides a measure of treatment credibility and expectancy. Treatment credibility refers to the patient's beliefs about the logic of the intervention whereas treatment expectancy refers to the patient's beliefs about how much they think they might improve [48]. It has been shown that a patient's expectations and their initial beliefs about the credibility of a given pain treatment affect treatment outcome [48]. Therefore, if differences in patient beliefs are unequal between groups in this trial, the observed outcome might not be attributed to the independent variable (i.e. real dry needling).

The Credibility/Expectancy Questionnaire consists of 6 items, 3 of which are related to the credibility factor and 3 are related to the expectancy factor. For each item, participants will be asked to rate the credibility of the treatment and their expectations on a 9-point Likert scale. High scores on the scale indicate the participant thinks the treatment is credible and either thinks and/or feels the treatment will result in substantial improvement in their symptoms. The Credibility/Expectancy Questionnaire has been shown to have good internal consistency and test-retest reliability [47]. A modified version of the Credibility/Expectancy Questionnaire has been used previously to evaluate the credibility of real dry needling versus sham dry needling for patients with whiplash associated pain [28]. The CEQ will be administered after the first treatment.

### Sample size

Eighty participants (i.e. 40 per group) with plantar heel pain (who satisfy the inclusion and exclusion criteria) will be recruited. An initial prospective sample size calculation estimated that 76 participants will provide 80% power to detect a minimally important difference of 13 points in the pain domain of the FHSQ [49] with a standard deviation of 20 points and an alpha set at 0.05. This sample size will also be sufficient to detect a minimally important difference of 19 mm for the other primary outcome measure, 'first-step' pain measured on a visual analogue scale.

### Statistical Analysis

Statistical analysis will be performed using the SPSS (SPSS Corp, Chicago III, USA) software. If the participant has bilateral symptoms, data from the most painful side will be recorded and analysed [50]. Data analysis will follow the intention-to-treat principle using all randomised participants [51] and missing data will be handled using a modified group mean substitution method [52]. This method involves substituting the missing data value with the mean baseline score plus the difference between the mean baseline and mean follow-up score for that particular group. Standard tests to assess continuous data for normal distribution will be used and transformation carried out if required.

Demographic and anthropometric characteristics (gender, age, mass, height, body mass index, waist to hip circumference ratio, sporting activities, foot posture using the FPI and the number of MTrPs located in the soleus, abductor hallucis, flexor digitorum brevis and quadratus plantae) will be determined for each treatment group. Summary statistics will also be calculated for duration of symptoms and side affected (left, right or both).

Outcomes measured at 2, 4, 6 and 12 weeks will be analysed. A linear regression approach to ANCOVA will be used to assess for differences in continuous outcomes between the two groups [53]. Appropriate non-parametric statistical tests will be used for outcomes that are nominal and ordinal scaled. The p-value will be set at 0.05.

## Discussion

Plantar heel pain is a common complaint that has been found to have a negative impact on foot specific and health-related quality of life [7]. Despite dry needling being increasingly used for musculoskeletal pain [10], there is a paucity of research determining its efficacy. Therefore, the primary aim of this study is to evaluate whether trigger point dry needling is more effective in reducing plantar heel pain than a sham dry needling intervention. The secondary aim is to evaluate whether dry needling results in changes to foot function; general foot health; depression, anxiety and stress and health-related quality of life in people with plantar heel pain.

In this study, the effectiveness of dry needling will be evaluated using a control comparison. The choice of control was influenced by the research question. As we are attempting to determine if dry needling has any treatment effect, the control needed to be an intervention that was indistinguishable, and applied using the same method, as the real intervention (i.e. real dry needling).

Other control options included a no treatment or waiting list or standard therapy (in a trial where dry needling plus standard therapy is compared with standard therapy alone) [54]. A waiting list control was not chosen as the non-specific effects of dry needling are not controlled. A standard therapy control was discounted because it is difficult to separate the influence of dry needling from other therapies used (e.g. orthoses, taping, stretching, strengthening). In addition, participants in the standard therapy group might be disappointed when they realise they will not receive the intervention of interest [54]. This state, called resentful demoralisation [55], results in bias in clinical trials.

There are no guidelines regarding the use of dry needling for plantar heel pain. Therefore, leading up to our trial, we conducted a consensus study (using a modified Delphi process) over 3 rounds to determine a protocol that was pragmatic and closely resembles clinical practice (*unpublished data: Cotchett MP, Landorf KB, Munteanu SE, Raspovic AM: Consensus for dry needling for plantar heel pain (plantar fasciitis): a modified Delphi study. Manuscript submitted for publication*). Thirty experts, from 10 countries, indicated their level of agreement on specific items relating to the use of dry needling for plantar heel pain including: the treatment rationale; dry needling details; brand of acupuncture needle; muscles dry needled; depth of insertion; number of needle insertions per muscle; needle retention time; manual manipulation of the needle; type of response elicited; and treatment regimen. The outcome of the Delphi study was that a consensus driven dry needling protocol for plantar heel pain was established.

The final protocol established by consensus underwent one modification after Round 3 without approval from the Delphi participants. We removed the posterior tibial muscle as a structure that might be assessed and if appropriate, dry needled. This was in response to a recent study recommending that needle insertion into the tibialis posterior only be undertaken using ultrasound guidance due to close proximity of neurovascular bundles [56]. Further, another study has shown that manual localisation of the posterior tibial muscle using anatomical landmarks had a failure rate of 88% [57].

In conclusion, this study is the first randomised controlled trial to evaluate the effectiveness of dry needling for plantar heel pain. The trial will be reported in accordance with the CONSORT and STRICTA group statements. Recruitment for the trial will begin in February 2011.

## Competing interests

KBL is a Deputy Editor and SEM is an Associate Editor of the Journal of Foot and Ankle Research. It is journal policy that editors are removed from the peer review and editorial decision-making processes for manuscripts they have co-authored.

## Authors' contributions

MC, KBL, SEM and AMR conceived the idea and designed the trial protocol. MC obtained funding for the study. All authors designed the trial protocol and drafted the manuscript. All authors read and approved the final manuscript.

## Supplementary Material

Additional File 1 **A demonstration of the real dry needling technique to be used in this trial**. Additional File 1 contains a demonstration of dry needling of the abductor hallucis muscle. Click here for file

Additional File 2 **A demonstration of the sham dry needling technique to be used in this trial**. Additional File 1 contains a demonstration of sham dry needling of the peroneus longus muscle. Click here for file

Additional File 3 **Explanation of the treatment procedure to participants**. Additional File 3 contains an explanation of the treatment procedure given to the participant prior to its commencement. Click here for file
