# Supplementary material for: Effectiveness of trigger point dry needling for plantar heel pain: study protocol for a randomised controlled trial
Source: J Foot Ankle Res. 2011 Jan 23;4:5. doi: 10.1186/1757-1146-4-5 (PMC3035595; doi:10.1186/1757-1146-4-5)
Supplement: Additional File 3 — Explanation of the treatment procedure to participants. Additional File 3 contains an explanation of the treatment procedure given to the participant prior to its commencement. [file 1757-1146-4-5-S3.DOC]

**Effectiveness of trigger point dry needling for plantar heel pain: study protocol for a randomised controlled trial**

Matthew P Cotchett^1, 2 §^, Karl B Landorf ^1, 2^, Shannon E Munteanu^1, 2^, Anita M Raspovic^1, 2^

**Additional File 2**

Title: Explanation of the treatment procedure to participants.

Description: Prior to the commencement of treatment, the following explanation will be given to participants:

*“I will first attempt to locate trigger points within four muscles of the lower limb including the soleus, quadratus plantae, flexor digitorum brevis and abductor hallucis muscles (a poster will be used to show the location of each muscle). An assessment will also be conducted to identify trigger points within other muscles of the lower limb that might impact on the muscles just listed (a poster will be used to show the location of additional muscles). I will locate a trigger point by moving my fingers along the length of the muscle until I reach an area where there is a taut band within the muscle. At this point I will press firmly on that point and ask you if it produces pain locally or in other areas. With a pen I will mark all the tender areas that are to be treated”.*

To commence the trigger point dry needling procedure a standardised explanation will be given to both groups.

*“To commence the procedure I will remove the acupuncture needle from its packaging. The guide tube housing the acupuncture needle will be positioned against your skin overlying the trigger point. I will then tap the needle against your skin and move the needle up and down carefully (the procedure will be demonstrated to the participant). As I move the needle I will ask you to describe any sensations you might be experiencing close to, or away from the site of the needle and whether or not the sensations are tolerable. I will move the needle up and down for approximately thirty seconds. I will then leave the needle to rest for five minutes. Each trigger point will be treated one at a time. If you would like the treatment to stop at any point, please let me know”*
